# The Role of Convective Up‐ and Downdrafts in the Transport of Trace Gases in the Amazon

**DOI:** 10.1029/2022JD037265

**Published:** 2022-09-22

**Authors:** Roman Bardakov, Radovan Krejci, Ilona Riipinen, Annica M. L. Ekman

**Affiliations:** ^1^ Department of Meteorology Stockholm University Stockholm Sweden; ^2^ Bolin Centre for Climate Research Stockholm University Stockholm Sweden; ^3^ Department of Environmental Science (ACES) Stockholm University Stockholm Sweden

**Keywords:** deep convection, trace gas transport, convective updraft, convective downdraft

## Abstract

Deep convective clouds can redistribute gaseous species and particulate matter among different layers of the troposphere with important implications for atmospheric chemistry and climate. The large number of atmospheric trace gases of different volatility makes it challenging to predict their partitioning between hydrometeors and gas phase inside highly dynamic deep convective clouds. In this study, we use an ensemble of 51,200 trajectories simulated with a cloud‐resolving model to characterize up‐ and downdrafts within Amazonian deep convective clouds. We also estimate the transport of a set of hypothetical non‐reactive gases of different volatility, within the up‐ and downdrafts. We find that convective air parcels originating from the boundary layer (i.e., originating at 0.5 km altitude), can transport up to 25% of an intermediate volatility gas species (e.g., methyl hydrogen peroxide) and up to 60% of high volatility gas species (e.g., n‐butane) to the cloud outflow above 10 km through the mean convective updraft. At the same time, the same type of gases can be transported to the boundary layer from the middle troposphere (i.e., originating at 5 km) within the mean convective downdraft with an efficiency close to 100%. Low volatility gases (e.g., nitric acid) are not efficiently transported, neither by the updrafts nor downdrafts, if the gas is assumed to be fully retained in a droplet upon freezing. The derived properties of the mean up‐ and downdraft can be used in future studies for investigating convective transport of a larger set of reactive trace gases.

## Key Points

- Typical properties of deep convective up‐ and downdrafts in the Amazon are obtained based on a sample of simulated clouds
- Up to 17% of the lower boundary layer air can reach the upper troposphere during a deep convective cloud event
- Intermediate and high volatility trace gases can be efficiently transported from the mid‐troposphere to the boundary layer with downdrafts

## 1 Introduction

Deep convection is the main transport pathway connecting planetary boundary layer (BL) and the upper troposphere in tropics. The deep convective clouds also redistribute gaseous and particulate matter within the tropical troposphere. This influences atmospheric chemistry as well as background concentrations of aerosol particles (e.g., Barth et al., 2015, 2016; C. Wang et al., 1995; Chatfield & Crutzen, 1984; Cooper et al., 2006; Dickerson et al., 1987; Twohy et al., 2002, and others). Twohy et al. (2002) pointed out that deep convective clouds can rapidly inject a gas from the BL into the upper troposphere (UT) where subsequent chemical and physical processing can lead to in‐situ new particle formation (NPF). Indeed, numerous flight campaigns have observed high abundances of small aerosol particles in the UT in the vicinity of tropical deep convection (e.g., Andreae et al., 2018; Clarke et al., 1999; Krejci et al., 2003; Twohy et al., 2002; Weigel et al., 2011).

It is well known that oxidized volatile organic compounds (VOCs) can enhance NPF in the presence of sulfate (Riccobono et al., 2012). Potentially, the oxidized VOCs can also trigger NPF on their own, in particular in the UT where temperatures are relatively cold (Frege et al., 2018). The Amazon region is of particular interest in this context due to the high abundance of biogenic organic compounds in the BL that could survive transport to the cloud outlfow region (Bardakov et al., 2021) and eventually play an important role for NPF (Bianchi et al., 2016). Once nucleated and grown by coagulation and condensation, the newly formed particles can be transported back to the BL through large‐scale subsidence, for example, in the outer parts of Hadley cell, and influence low‐level clouds, radiation, and climate (Clarke et al., 1999; Williamson et al., 2019). Recently, a transport pattern connecting the tropical free troposphere with the BL through cloud‐scale downdrafts was also identified over the Amazon (J. Wang et al., 2016; Machado et al., 2021), underlining that it is not only the updrafts that are important for the vertical redistribution of gases and aerosols within deep convective clouds. Furthermore, detrainment and entrainment of air along the boundaries of a deep convective cloud may under certain conditions be a source of gases and aerosols from the BL to the mid‐troposphere (Bourgeois et al., 2016; Engström et al., 2008) or result in transport of aerosols from the mid‐troposphere to the UT (Fridlind et al., 2004).

The complex patterns of redistribution of air along with the accompanying cloud microphysics must be captured when modeling convective gas transport in order to estimate sources and sinks of the gases correctly. This is usually achieved by performing simulations with high‐resolution cloud‐resolving models (CRMs) (see e.g., Barth et al., 2007; Bela et al., 2018; Ekman et al., 2008; Murphy et al., 2015). However, only a limited number of chemical spieces can typically be modeled with this approach due to high computational cost. Bardakov et al. (2020) developed a computationally efficient framework to estimate the transport of trace gases from the boundary layer into the upper troposphere through an individual cloud. The framework makes use of high‐resolution air parcel trajectory output from a CRM simulation. Bardakov et al. (2020) modeled the vertical transport of trace gases with different volatilities based on two deep convective cloud cases (one idealized and one from the Deep Convective Clouds and Chemistry (DC3) field campaign (Barth et al., 2016)). While the focus of Bardakov et al. (2020) was to understand the impact of the molecular properties of the gases driving their transport, the general trends over a large number of possible trajectories as well as the potential impact of downdrafts were not explored. Therefore, we here expand on the analysis of Bardakov et al. (2020), and use the same CRM to simulate a large ensemble of air parcel trajectories for a number of deep convective clouds over the Amazon. We calculate the statistical likelihood for an air parcel originating in one part of the troposphere to reach other parts during a convective event. From the ensemble, we derive a novel data set describing two mean air parcel trajectories representing average convective up‐ and downdrafts in the Amazon. These average trajactories are then used together with the box model described in Bardakov et al. (2020) to calculate the up‐ and downward transport of three idealized non‐reactive trace gases of different volatility, as well as to estimate the role of the gas condensation sink and turbulent mixing during the transport.

## 2 Methods

The air parcel trajectories were extracted from simulations using the MIT‐MISU Cloud Aerosol model (MIMICA). MIMICA is a CRM that solves the anelastic, nonhydrostatic governing equations in three dimensions (Savre et al., 2014). The microphysics scheme used in the current version of MIMICA predicts the mass mixing ratio of cloud precipitation and condensate particles, which in turn are divided into water and ice hydrometeors depending on the modeled temperature (Grabowski, 1998). Both water droplets and ice particles are assumed to have a simple spherical shape. Cloud condensate particles have a monodisperse size distribution and follow the flow of air without precipitating, while precipitating particles follow a Marshall‐Palmer distribution. The mean terminal velocity of the latter particles is estimated from the size distribution and the particle mass mixing ratio within the grid box. Turbulence is parameterized following a Smagorinski‐Lilly approach (Lilly, 1962; Smagorinsky, 1963).

Atmospheric soundings from the University of Wyoming sounding data archive (http://weather.uwyo.edu/upperair/sounding.html) were used to initialize the MIMICA simulations. The soundings were retrieved over Manaus (Latitude: −3.1, Longitude: −60.0) during the wet season from April 1 until 14 April 2020 at 00 and 12 UTC (excluding one unavailable sounding from April 3 at 00 UTC, i.e., in total 27 soundings). The simulations were performed for 2 hr over a domain covering 200 × 200 × 20 km^3^, with 512 × 512 grid points in the horizontal direction and 200 grid points in the vertical (this gives a horizontal and vertical resolution of 390 and 100 m, respectively). To mimic deep convective clouds that have a high potential of transporting chemical species into the upper troposphere, the simulations were initiated by a warm bubble perturbation with a maximum temperature difference of 5 K that was set in the middle of the bubble and then varied as a cosine squared toward the edge. The center of the bubble was situated at *x* = 100 km, *y* = 100 km and *z* = 0 km. The radius of the bubble was 2 km in the vertical direction and 20 km in the horizontal directions.

The initial air parcel positions were set to the following altitudes: 0.5, 1.5, 2.5, 3.5, 5, 7, 9, and 11 km, respectively, with 400 air parcels released in each layer, that is, every cloud case generated 3,200 air parcel trajectories. The initial parcel positions were distributed uniformly in the horizontal direction within a rectangular area defined by the radius of the perturbation bubble (i.e., each side of the rectangle is twice the horizontal radius of the bubble). When analyzing the output, the simulated air parcel trajectories were divided into three height bins: 0–3, 3–10, and >10 km. These layers roughly represent the lower troposphere (or the BL), the middle troposphere, and the UT (including the deep convective cloud outflow), respectively. A distinct mixing layer could not always be identified in the sounding profiles, most likely due to the time of the day when the soundings were retrieved. Therefore, following Zimmerman et al. (1988), we assume that the layer below 3 km roughly represents a well‐mixed lower troposphere (including the surface mixing layer and a shallow convective cloud layer), and hereafter refer to this part of the troposphere as the BL. Since the deep convective cloud anvil typically reaches higher altitudes than 10 km (Takahashi et al., 2017) we define the cloud outflow region in the UT to be above 10 km. In other words, trajectories with initial positions at 0.5, 1.5, and 2.5 km represent the BL, those starting at 3.5, 5, 7, and 9 km represent the middle troposphere and those starting at 11 km the UT.

We focused our analysis on cloud cases that triggered deep convection and that injected a non‐negligible amount of air into the UT. More specifically, we only selected cases where at least 1% of the simulated BL parcels reached an altitude of 10 km or more. We grouped air parcels that reached a certain altitude bin (BL, middle troposphere or UT) at the end of the simulation and calculated key characteristics such as the mean and standard deviation of altitude, temperature, reversed turbulent mixing time scale, vapor, and condensate and precipitation concentrations at each time step. The reversed turbulent mixing scale was defined as the ratio between the sub‐grid turbulent diffusion coefficient and the squared length scale of the simulation grid, see also Equation 22 in Bardakov et al. (2020)). In addition to the main analysis of the air parcels for the deep convective clouds in the Amazon, a sensitivity test for the parcels originating at the four lower altitudes (i.e., at 0.5, 1.5, 2.5, and 3.5 km) with a maximum bubble perturbation of 3 K (as compared with the 5 K perturbation in the base case) was performed.

To study the convective transport of non‐reactive trace gases within the simulated deep convective clouds, we used the box model described in detail in Bardakov et al. (2020). The model solves a set of linear equations that describe and assess the change in the gas concentration due to cloud processes, including condensation and evaporation to/from hydrometeors, removal by precipitation, and turbulent mixing. We assumed that any gas is fully retained in a droplet upon freezing. We examined three hypothetical trace gases that differ by their volatility: low, intermediate and high. The volatilities were defined using the effective gas saturation vapor pressure, $P_{\mathrm{eff}}^{\theta }$, with values 5 ⋅ 10^7^, 5 ⋅ 10^5^ and 5 ⋅ 10^1^ Pa, respectively, and enthalpy of vapourization, Δ*H*, with values 25, 55, and 85 kJ mol^−1^, respectively, according to Bardakov et al. (2020). According to the given definitions and neglecting chemical reactions, our high volatile gas could be associated with, for example, n‐butane, the intermediate volatile gas with methyl hydrogen peroxide and the low volatile gas with nitric acid.

## 3 Results

### 3.1 General Behavior of the Simulated Air Parcels

**Figure 1:**
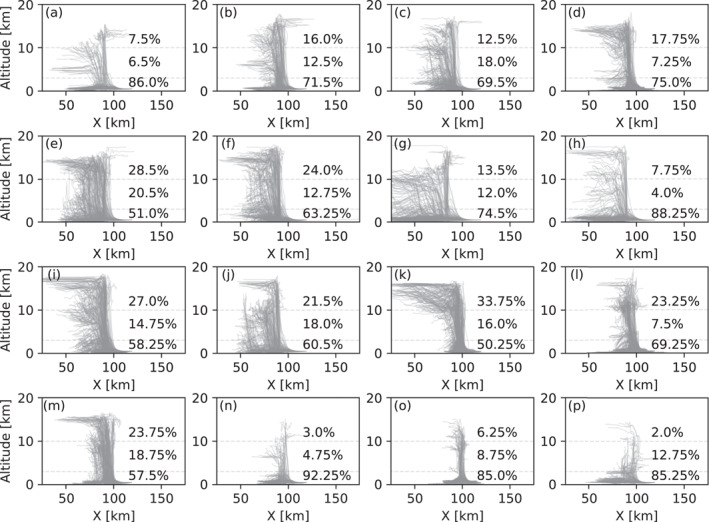
Trajectories for air parcels initially located at 0.5 km altitude for all cases where deep convection was triggered. Top, middle and bottom numbers in each subfigure show percentages of parcels that reached the >10, 3–10, and 0–3 km height bins, respectively. Simulation time is 2 hr.

From the CRM simulations, deep convection (according to the criterion defined in Section 2) was produced from 16 thermodynamic profiles out of 27. Based on the air parcel trajectories of the triggered cloud cases we infer that the air parcels from the lowest level of the BL (i.e., 0.5 km) usually reach the UT within 1 hr of simulation. The cloud anvil is formed within the next hour. Figures 1a–1p shows the trajectories for air parcels that initially were situated at 0.5 km height for each of the 16 deep convective cloud cases after 2 hr of simulation. An analysis of the final air parcel altitudes shows that in all of the cloud cases, at least 50% of the air parcels starting at 0.5 km do not leave the BL and stay below 3 km altitude. Instead, they remain around the initial altitude or sink toward the surface. Up to 20% of the parcels end up between 3 and 10 km height in the middle troposphere and up to 33% of the parcels reach the UT at 10 km or above. In other words, the transport of air parcels from the surface to the UT is in general more efficient than the transport to the middle troposphere.

The transport efficiency to the UT region is, however, sensitive to the magnitude of the initial warm bubble perturbation. When the maximum initial bubble perturbation is lowered from 5 to 3 K, the convection becomes weaker and only around 7% of the air parcels originating from 0.5 km height reach the UT. In some cases, convection is not even triggered (see Figures S1 and S2 in Supporting Information S1).

**Figure 2:**
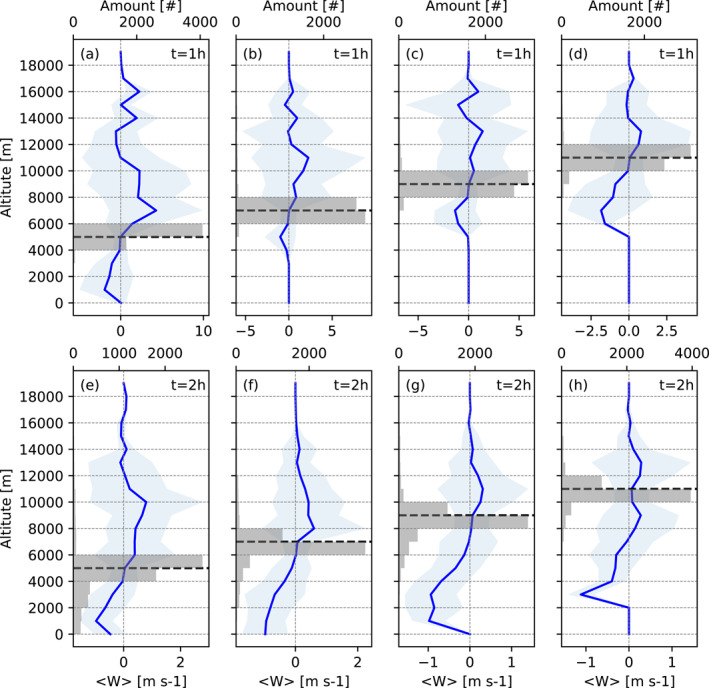
Vertical distribution of mean vertical velocity for all parcels initially located at *z*_0_ = 5, 7, 9, and 11 km height for all deep convective cloud cases (blue line) at 1 h (upper panel) and 2 h (lower panel) of simulation. Shaded light blue area shows the standard deviation of the velocities. The gray horizontal bars show cumulative amount of air parcels in the bins corresponding to the mean vertical velocity. Black dashed line indicates initial parcel location.

Figures 2a–2h shows the mean vertical velocities as a function of altitude for all the parcels initially located in the middle and upper troposphere at 5, 7, 9, and 11 km altitude after 1 and 2 hr of simulation (upper and lower panel, respectively). After 1 hr of simulation most parcels stay around their original level as the result of small mean vertical velocity and small deviation of the air parcel velocity from the mean (the positions are indicated by gray horizontal bars in Figure 2). This result shows that downward (as well as upward) motion from the middle troposphere is uncommon at the early stages of deep convective cloud evolution. At *t* = 2 hr, however, a much larger number of parcels (see gray horizontal bars in Figure 2) have negative vertical velocities. Parcels initially located between 5 and 9 km show a distinct tendency to propagate toward the BL at this later stage of deep convective cloud development. In contrast, only a small number of parcels initially located in the UT (at 11 km height) have a negative vertical velocity (see Figures 2d and 2h). Nevertheless, our simulations indicate that up to 10% of the air parcels still can be transported from the UT to the middle troposphere during the 2 hr of simulation.

**Figure 3:**
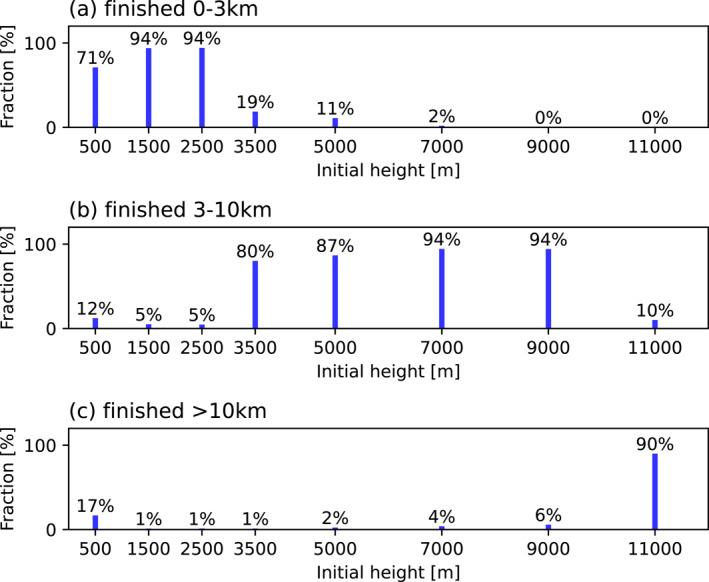
Fractions of parcels that end up within a specific height bin (BL: 0–3 km, middle troposphere: 3–10 km and UT: >10 km, which represents the convective cloud outflow) after 2 hr of simulations as a function of the initial parcel altitude.

Figures 3a–3c shows the fraction of parcels that end up within the BL (0–3 km), middle troposphere (3–10 km) or UT (>10 km) bins as a function of the initial parcel altitude after 2 hr of simulation for all deep convective cloud cases. A majority of the parcels do not leave their original altitude bin and their transport is driven mainly by horizontal advection. A small, but still substantial, fraction of air parcels undergo distinct vertical transport across altitude bins. On average, 17% of the parcels originating at 0.5 km height reach the cloud outflow and 12% end up in the middle troposphere. A substantially lower fraction of BL air reach the middle and upper troposphere from 1.5 to 2.5 km altitude (∼5% and ∼1%, respectively). Thus, our simulations indicate that it is the air from the lowest part of the BL, that is, the mixing layer above the rain forest, that is most efficiently transported into the upper troposphere over the Amazon region. However, this result may be sensitive to the location of the initial buoyancy perturbation, which here was set to be largest at the surface. Parcels originating just above the BL, that is, at 3.5 and 5 km, mainly stay at their initial altitude or move downwards. Approximately 11%–19% of the air parcels from the lower free troposphere end up in the BL after 2 hr of simulation, showing that free troposheric air can be transported to the boundary layer by convectively‐driven downdrafts. Only 4% and 6% of the parcels initially located at 7 and 9 km, respectively, reach the UT above 10 km. The vast majority of the parcels (87%–94%) stay in the middle troposphere, with a tendency to sink to the lower altitudes but without reaching the BL within 2 hr of simulation time. A similar behavior is observed for parcels starting in the UT. These parcels mainly stay in the UT (90%) and only a small fraction (10%) of the air parcels sink to the middle troposphere, while no parcel reaches the BL within the simulation time. An example of the trajectory distribution for one of the cases (9 April 2020, 12 UTC) is depicted in three dimensions in Figures S3–S9 in Supporting Information S1 to give an idea of how air parcels from different initial altitudes are redistributed in space after 2 hr of simulation. In general the simulated trajectories closely replicate well‐known dynamical patterns of deep convection, such as convective‐scale up‐ and downdrafts as described for example, by Houze Jr. and Betts (1981).

#### 3.1.1 Mean Up‐ and Downdraft Trajectories

**Figure 4:**
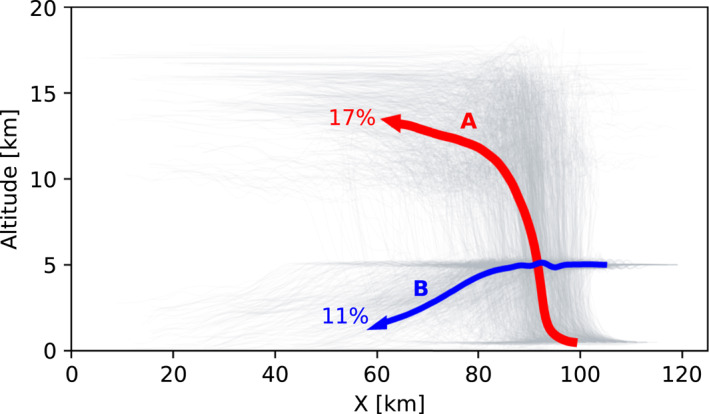
Mean trajectories of the parcels starting at 0.5 km altitude and finishing at >10 km that represent the updraft (A, red) and those starting at 5 km altitude and finishing <3 km that represent the downdraft (B, blue). Percentages indicate the corresponding fractions of parcels contributing to the up‐ or downdraft. Gray trajectories indicate individual up‐ and downdraft air parcels used for averaging. Simulation time is 2 hr.

The mean trajectories of the air parcels that start at 0.5 km altitude and then reach the cloud outflow above 10 km (convective updraft) as well as the ones that start in the middle troposphere at 5 km and then are transported downwards to the BL (convective downdraft) represent large displacements of air within a deep convective cloud and are further analyzed and shown in Figure 4. The extent of the horizontal propagation of the downdraft before it reaches the BL is ∼40 km.

**Figure 5:**
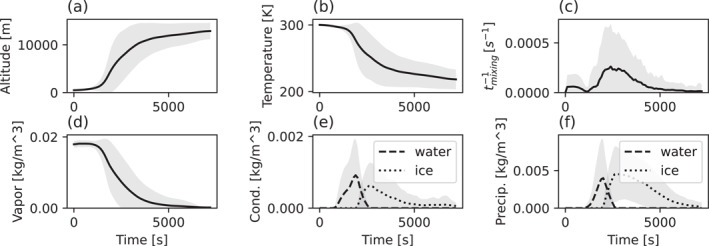
Mean and standard deviation of the altitude, temperature, reversed timescale of turbulent mixing, water vapor content, cloud condensate content and cloud precipitation content as a function of time for the updraft (A in Figure 4).

Figure 5 shows the mean and standard deviation of the altitude, temperature, reverse timescale of turbulent mixing, water vapor content, cloud condensate content and cloud precipitation content as a function of time for the mean convective updraft (trajectory A in Figure 4). The parcels in the updraft experience highly dynamic changes in temperature, pressure, water vapor concentration, and hydrometeor mass and phase. They are also exposed to different levels of turbulence during the transport. The most drastic changes occur within ∼1.5 hr of simulation when the air is advected to the cloud outflow. Afterward, all parameters reach a quasi‐stationary state displaying only small changes. The mean altitude reached by the parcels in the outflow after 2 hr of simulation is 12.8 ± 1.7 km and the corresponding temperature is 219 ± 14.7 K. Turbulent mixing is relatively high at the beginning of the simulation, when the parcels reside within the BL. It then decreases when the parcels reach the mid‐troposphere and reach a second maximum when the parcels approach the outflow. The maximum value for the reverse timescale of turbulent mixing, showing the maximum influence of turbulence, is reached at 2,400 s around 7 km altitude and is 0.0002 ± 0.0004 s^−1^. The values are close to zero after ∼5,000 s meaning that the air in the outflow is advected under low turbulence conditions. The maximum total hydrometeor mass concentration reached is 1 ± 1 g m^−3^ for condensate and 5 ± 5 g m^−3^ for precipitation particles at around ∼2,000 s of simulation. The mean hydrometeor and water vapor concentrations approach zero after ∼1.5 hr and by that time the cloud updrafts generally start to dissipate. In the DC3 case simulated in Bardakov et al. (2020) with the same model, the development of the cloud occurred faster with high amounts of precipitation observed already around *t* = 1,000 s of simulation. The average altitude reached by the parcels was around 11 km and the temperature around 225 K. The magnitudes of other variables were similar to those of the mean upward trajectory described above.

**Figure 6:**
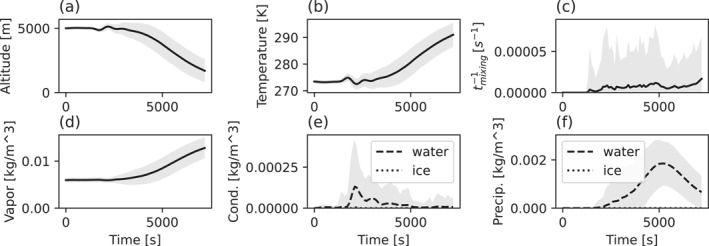
Same as in Figure 5 but for the downdraft (B in Figure 4).

The opposite behavior is to some extent observed for the air parcels that move downward during convection. Figure 6 shows the same time‐dependent parameters as in Figure 5 but for the mean convective downdraft (trajectory B in Figure 4). The downward motion starts after approximately 1 hr of cloud evolution and is associated with a simultaneous weakening of the updraft. As the average altitude decreases, there is a corresponding increase in the average temperature from ∼274 K to ∼290 ± 5 K and water vapor content from ∼6 to ∼12 ± 4 g m^−3^. The parcels generally experience less turbulence when they propagate downward compared to when they move upward; the reverse time scale of turbulent mixing is usually more than one order of magnitude higher within the updraft compared to within the downdraft. Since the parcels sink from 5 km where the temperature is above 273 K, no ice is present in the system and only water droplets exist. The amount of condensate water peaks in the downdraft around approximately 2,000 s, while precipitation water is the highest (∼2 ± 1 g m^−3^) at later stages of convection due to the dissipating updrafts and in‐situ condensation.

### 3.2 Gas Transport Within the Mean Up‐ and Downdraft

**Figure 7:**
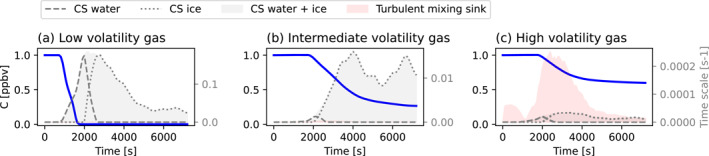
(a) Concentration of a low, (b) intermediate and (c) high volatile non‐reactive gas as a function of time along the mean updraft trajectory. Gray shaded area shows the condensation sink, red shaded area shows the turbulent mixing sink. Dashed and dotted lines denote condensation sink attributed to water and ice hydrometeors, respectively.

The average air parcel properties presented in Section 3.1.1 allow us to calculate the condensation sink (CS), the mixing time scale, and the fraction of a non‐reactive trace gas that can be transported on average from the BL into the UT (using the same methodology as in Bardakov et al., 2020). Figure 7 shows the time evolution of a hypothetical low‐, intermediate‐ and high volatile gas along the mean outflow trajectory presented in Figure 5. The initial gas concentration is assumed to be 1 ppbv. The CS for the low volatile gas reaches ∼0.16 s^−1^ at *t* = 2,000 s which leads to a complete removal of the gas. The quick condensational removal is caused by the high effective saturation vapor pressure of 5 ⋅ 10^7^ Pa in combination with the decrease in temperature along the air parcel trajectory. Under these conditions, nearly all the gas is taken up by the available hydrometeors in order to reach the equilibrium between the gaseous and condensed states. The CS for the gas with intermediate volatility reaches values of ∼0.01 s^−1^ and the gas is only partially removed. About 0.3 ppbv of the gas is transported into the UT, where an equilibrium between the gas in the vapor phase and the CS is reached. The transport of the intermediate volatility gas is sensitive to the specific value of the saturation vapor pressure, the enthalpy of vapourization and the temperature within the system. The CS for the high volatile gas is small compared to the other two gases and reaches a maximum value of ∼0.00005 s^−1^. In this case, it is mainly the turbulent mixing (shown by the turbulent mixing time scale which peaks at ∼0.0002 s^−1^) that causes a noticeable decrease in the gas concentration which reaches ∼0.6 ppbv in the UT, assuming zero concentration of the gas in the background. Bardakov et al. (2020) reported similar modeling results for convective transport of some common chemical compounds from the BL into the UT based on a convective case from the DC3 field campaign. They showed that during the transport, low‐volatile hydrogen peroxide (H_2_O_2_) is completely scavenged, intermediate‐volatile methyl hydrogen peroxide (CH_3_OOH) undergoes partial scavenging of up to 60% from its BL concentration and the high‐volatile n‐butane is efficiently transported without any scavenging with the concentration reduced by 40% only due to mixing with the out‐of‐cloud air.

**Figure 8:**
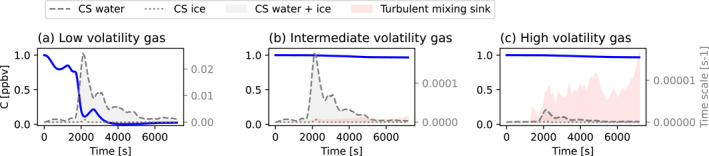
Same as in Figure 7 but along the mean downdraft trajectory.

Figure 8 shows the time evolution of a hypothetical low‐, intermediate‐ and high volatile gas along the mean trajectory of air parcels propagating from 5 km down to below 3 km (presented in Figure 6). The CS for the low volatile gas reaches a maximum of ∼0.02 s^−1^ at *t* = 2,000 s and then decreases until the end of the simulation, eventually leading to a near‐complete removal of the gas before it reaches the BL. The CS for the intermediate volatility gas reaches a maximum of ∼0.00017 s^−1^. This sink is not large enough to lead to any significant concentration decrease during the transport toward the BL. The CS and the turbulent mixing time scale are small for the high volatile gas, ∼0.00001 s^−1^ or less, and there is no substantial change in the gas concentration along the trajectory.

## 4 Summary and Conclusions

We have used the CRM MIMICA and the framework of Bardakov et al. (2020) to simulate a set of deep convective clouds in the Amazon, to describe the redistribution of air parcels within the deep convective clouds, and to calculate the fraction of a non‐reactive trace gas species that can be transported from the BL to the UT and from the middle troposphere down to the BL.

We found that air parcels located in the lowest layers of the BL had the highest probability of reaching the UT. Around 17% of all parcels initially located at 0.5 km height ended up at altitudes of 10 km or higher during the 2 hr of simulation. Air parcels starting in the upper layers of the BL (1.5 and 2.5 km) had a substantially lower probability of reaching the UT (about 1%). This result suggests that deep convective inflow of trace gases or particles can generally be well‐characterized with observational data from low altitudes in the BL provided that the maximum temperature perturbation occurs near the surface. We also analyzed the downdrafts within the simulated deep convective clouds. Approximately 19% and 11% of the air parcels located in the lower middle troposphere at 3.5 and 5 km height, respectively, reached the BL as a result of coherent and persistent convective downdrafts. This result is well in line with that of J. Wang et al. (2016) who suggested that convective downdrafts can be responsible for transporting air and Aitken mode aerosol particles from the free troposphere to the BL. This result underlines the importance of understanding transport of gases and aerosols not only within the updrafts but also within the deep convective downdrafts since these gases and aerosols may affect boundary layer chemistry, the availability of cloud condensation nuclei, and low‐level cloud formation. Air parcels located at 7–11 km height showed a tendency to move downward during the convective event, but the downward motion was weak and the parcels did not reach the BL within the 2 hr of simulation. We acknowledge, however, that larger‐scale compensating subsidence motions associated with deep convection (as discussed in e.g., Yanai et al., 1973) can lead to additional transport from the UT to lower altitudes on longer timescales (relatively to those considered in the current study). Future studies should examine how the local‐scale transport compares to the transport on a larger scale as well as the sensitivity of the transport to the vertical location of the initial buoyancy perturbation. It would also be useful to examine if and how other cloud microphysics schemes and related parametrizations affect the vertical redistribution of air.

Using derived typical upward and downward transport pattern from our simulations, we modeled the transport of trace gases with different volatility. The relative role of gas condensation and turbulent mixing during the transport was also evaluated. In the convective updraft, we found that turbulent mixing had the highest influence on the high‐volatility gas, reducing its concentration by 40%. Condensation was the most important sink for the low and intermediate volatility compounds. It led to a complete and quick removal of the low volatile gas from the system and to reduction of the intermediate volatile gas concentration by ∼75%. In the convective downdraft, similar to the updraft, the low‐volatility gas was almost completely removed by condensational uptake onto cloud hydrometeors, while the gases with intermediate and high volatility could be transported from the middle troposphere to the BL without substantial losses. Turbulent mixing had a negligible effect on all gases in the downdrafts. The modeled behaviour agrees well with our general understanding of gas interaction with clouds under different thermodynamic conditions (Seinfeld & Pandis, 1998) and the results presented earlier by (Bardakov et al., 2020).

The gas system used in this study is idealized, but convective transport of a more complex multi‐component gas and particle mixture can be modeled in the future using the derived cloud trajectory output. Such an approach will give us an improved picture of the interplay between cloud dynamics and gas chemistry and physics. It will also allow us to estimate the transport of specific atmospheric compounds to high altitudes and evaluate their significance for example, the recently discovered NPF mechanisms in the upper troposphere (M. Wang et al., 2022).

## Supporting information

Supporting Information S1

## Data Availability

Air parcel trajectories data generated by the modified MIMICA version 5 model code is available online (Bardakov, Krejci, et al., 2022). The trajectory framework code (Bardakov, Riipinen, et al., 2022) and MIMICA code (Bardakov, Savre, & Ekman, 2022) are available online.

## References

[jgrd58205-bib-0001] Andreae, M. O. , Afchine, A. , Albrecht, R. , Holanda, B. A. , Artaxo, P. , Barbosa, H. M. , et al. (2018). Aerosol characteristics and particle production in the upper troposphere over the Amazon basin. Atmospheric Chemistry and Physics, 18(2), 921–961. 10.5194/acp-18-921-2018

[jgrd58205-bib-0002] Bardakov, R. , Krejci, R. , Riipinen, I. , & Ekman, A. M. L. (2022). Contain Air parcel trajectories dataset based on modeling of 16 deep convective clouds in the Amazon [Dataset]. Zenodo. 10.5281/zenodo.6624241

[jgrd58205-bib-0003] Bardakov, R. , Riipinen, I. , Krejci, R. , Savre, J. , Thornton, J. A. , & Ekman, A. M. (2020). A novel framework to study trace gas transport in deep convective clouds. Journal of Advances in Modeling Earth Systems, 12(5), e2019MS001931. 10.1029/2019ms001931

[jgrd58205-bib-0004] Bardakov, R. , Riipinen, I. , Krejci, R. , Savre, J. , Thornton, J. A. , & Ekman, A. M. L. (2022). Trajectory framework [software]. Zenodo. 10.5281/zenodo.7053973

[jgrd58205-bib-0005] Bardakov, R. , Savre, J. , & Ekman, A. M. L. (2022). Mimica [software]. Zenodo. 10.5281/zenodo.7053947

[jgrd58205-bib-0006] Bardakov, R. , Thornton, J. A. , Riipinen, I. , Krejci, R. , & Ekman, A. M. (2021). Transport and chemistry of isoprene and its oxidation products in deep convective clouds. Tellus B: Chemical and Physical Meteorology, 73(1), 1–21. 10.1080/16000889.2021.1979856

[jgrd58205-bib-0007] Barth, M. C. , Bela, M. , Fried, A. , Wennberg, P. , Crounse, J. , St. Clair, J. , et al. (2016). Convective transport and scavenging of peroxides by thunderstorms observed over the central us during DC3. Journal of Geophysical Research: Atmospheres, 121(8), 4272–4295. 10.1002/2015jd024570

[jgrd58205-bib-0008] Barth, M. C. , Cantrell, C. A. , Brune, W. H. , Rutledge, S. A. , Crawford, J. H. , Huntrieser, H. , et al. (2015). The deep convective clouds and chemistry (DC3) field campaign. Bulletin of the American Meteorological Society, 96(8), 1281–1309. 10.1175/bams-d-13-00290.1

[jgrd58205-bib-0009] Barth, M. C. , Kim, S.‐W. , Wang, C. , Pickering, K. , Ott, L. , Stenchikov, G. , et al. (2007). Cloud‐scale model intercomparison of chemical constituent transport in deep convection. Atmospheric Chemistry and Physics, 7(18), 4709–4731. 10.5194/acp-7-4709-2007

[jgrd58205-bib-0010] Bela, M. M. , Barth, M. C. , Toon, O. B. , Fried, A. , Ziegler, C. , Cummings, K. A. , et al. (2018). Effects of scavenging, entrainment, and aqueous chemistry on peroxides and formaldehyde in deep convective outflow over the central and southeast United States. Journal of Geophysical Research: Atmospheres, 123(14), 7594–7614. 10.1029/2018jd028271 PMC7427629 32802698

[jgrd58205-bib-0011] Bianchi, F. , Tröstl, J. , Junninen, H. , Frege, C. , Henne, S. , Hoyle, C. R. , et al. (2016). New particle formation in the free troposphere: A question of chemistry and timing. Science, 352(6289), 1109–1112. 10.1126/science.aad5456 27226488

[jgrd58205-bib-0012] Bourgeois, Q. , Ekman, A. M. , Igel, M. R. , & Krejci, R. (2016). Ubiquity and impact of thin mid‐level clouds in the tropics. Nature Communications, 7(1), 1–6. 10.1038/ncomms12432 PMC4992062 27530236

[jgrd58205-bib-0013] Chatfield, R. B. , & Crutzen, P. J. (1984). Sulfur dioxide in remote oceanic air: Cloud transport of reactive precursors. Journal of Geophysical Research, 89(D5), 7111–7132. 10.1029/jd089id05p07111

[jgrd58205-bib-0014] Clarke, A. D. , Eisele, F. , Kapustin, V. , Moore, K. , Tanner, D. , Mauldin, L. , et al. (1999). Nucleation in the equatorial free troposphere: Favorable environments during PEM‐tropics. Journal of Geophysical Research, 104(D5), 5735–5744. 10.1029/98jd02303

[jgrd58205-bib-0015] Cooper, O. R. , Stohl, A. , Trainer, M. , Thompson, A. , Witte, J. , Oltmans, S. , et al. (2006). Large upper tropospheric ozone enhancements above Midlatitude North America during summer: In situ evidence from the ions and mozaic ozone measurement network. Journal of Geophysical Research, 111(D24), D24S05. 10.1029/2006jd007306

[jgrd58205-bib-0016] Dickerson, R. R. , Huffman, G. , Luke, W. , Nunnermacker, L. , Pickering, K. , Leslie, A. , et al. (1987). Thunderstorms: An important mechanism in the transport of air pollutants. Science, 235(4787), 460–465. 10.1126/science.235.4787.460 17810340

[jgrd58205-bib-0017] Ekman, A. M. L. , Krejci, R. , Engström, A. , Ström, J. , de Reus, M. , Williams, J. , & Andreae, M. O. (2008). Do organics contribute to small particle formation in the Amazonian upper troposphere? Geophysical Research Letters, 35(17), L17810. 10.1029/2008gl034970

[jgrd58205-bib-0018] Engström, A. , Ekman, A. M. , Krejci, R. , Ström, J. , de Reus, M. , & Wang, C. (2008). Observational and modelling evidence of tropical deep convective clouds as a source of mid‐tropospheric accumulation mode aerosols. Geophysical Research Letters, 35(23), L23813. 10.1029/2008gl035817

[jgrd58205-bib-0019] Frege, C. , Ortega, I. K. , Rissanen, M. P. , Praplan, A. P. , Steiner, G. , Heinritzi, M. , et al. (2018). Influence of temperature on the molecular composition of ions and charged clusters during pure biogenic nucleation. Atmospheric Chemistry and Physics, 18(1), 65–79. 10.5194/acp-18-65-2018

[jgrd58205-bib-0020] Fridlind, A. M. , Ackerman, A. S. , Jensen, E. J. , Heymsfield, A. J. , Poellot, M. R. , Stevens, D. E. , et al. (2004). Evidence for the predominance of mid‐tropospheric aerosols as subtropical anvil cloud nuclei. Science, 304(5671), 718–722. 10.1126/science.1094947 15118158

[jgrd58205-bib-0021] Grabowski, W. W. (1998). Toward cloud resolving modeling of large‐scale tropical circulations: A simple cloud microphysics parameterization. Journal of the Atmospheric Sciences, 55(21), 3283–3298. 10.1175/1520-0469(1998)055<3283:tcrmol>2.0.co;2

[jgrd58205-bib-0022] Houze, R. A., Jr. , & Betts, A. K. (1981). Convection in gate. Reviews of Geophysics, 19(4), 541–576. 10.1029/rg019i004p00541

[jgrd58205-bib-0023] Krejci, R. , Ström, J. , de Reus, M. , Hoor, P. , Williams, J. , Fischer, H. , & Hansson, H.‐C. (2003). Evolution of aerosol properties over the rain forest in Surinam, South America, observed from aircraft during the lba‐Claire 98 experiment. Journal of Geophysical Research, 108(D18), 4561. 10.1029/2001jd001375

[jgrd58205-bib-0024] Lilly, D. K. (1962). On the numerical simulation of buoyant convection. Tellus, 14(2), 148–172. 10.3402/tellusa.v14i2.9537

[jgrd58205-bib-0025] Machado, L. A. T. , Franco, M. A. , Kremper, L. A. , Ditas, F. , Andreae, M. O. , Artaxo, P. , et al. (2021). How weather events modify aerosol particle size distributions in the amazon boundary layer. Atmospheric Chemistry and Physics Discussions, 21(23), 1–31. 10.5194/acp-21-18065-2021

[jgrd58205-bib-0026] Murphy, B. N. , Julin, J. , Riipinen, I. , & Ekman, A. M. L. (2015). Organic aerosol processing in tropical deep convective clouds: Development of a new model (CRM‐ORG) and implications for sources of particle number. Journal of Geophysical Research: Atmospheres, 120(19), 10–441. 10.1002/2015jd023551

[jgrd58205-bib-0027] Riccobono, F. , Rondo, L. , Sipilä, M. , Barmet, P. , Curtius, J. , Dommen, J. , et al. (2012). Contribution of sulfuric acid and oxidized organic compounds to particle formation and growth. Atmospheric Chemistry and Physics, 12(20), 9427–9439. 10.5194/acp-12-9427-2012

[jgrd58205-bib-0028] Savre, J. , Ekman, A. M. L. , & Svensson, G. (2014). Introduction to Mimica, a large‐eddy simulation solver for cloudy planetary boundary layers. Journal of Advances in Modeling Earth Systems, 6(3), 630–649. 10.1002/2013ms000292

[jgrd58205-bib-0029] Seinfeld, J. H. , & Pandis, S. N. (1998). Atmospheric chemistry and physics: From air pollution to climate change. John Wiley and Sons.

[jgrd58205-bib-0030] Smagorinsky, J. (1963). General circulation experiments with the primitive equations: I. The basic experiment. Monthly Weather Review, 91(3), 99–164. 10.1175/1520-0493(1963)091<0099:gcewtp>2.3.co;2

[jgrd58205-bib-0031] Takahashi, H. , Luo, Z. J. , & Stephens, G. L. (2017). Level of neutral buoyancy, deep convective outflow, and convective core: New perspectives based on 5 years of Cloudsat data. Journal of Geophysical Research: Atmospheres, 122(5), 2958–2969. 10.1002/2016jd025969

[jgrd58205-bib-0032] Twohy, C. H. , Clement, C. F. , Gandrud, B. W. , Weinheimer, A. J. , Campos, T. L. , Baumgardner, D. , et al. (2002). Deep convection as a source of new particles in the Midlatitude upper troposphere. Journal of Geophysical Research, 107(D21), 6‐1–6‐10. 10.1029/2001jd000323

[jgrd58205-bib-0033] Wang, C. , Crutzen, P. J. , Ramanathan, V. , & Williams, S. F. (1995). The role of a deep convective storm over the tropical Pacific Ocean in the redistribution of atmospheric chemical species. Journal of Geophysical Research, 100(D6), 11509–11516. 10.1029/95jd01173

[jgrd58205-bib-0034] Wang, J. , Krejci, R. , Giangrande, S. , Kuang, C. , Barbosa, H. M. , Brito, J. , et al. (2016). Amazon boundary layer aerosol concentration sustained by vertical transport during rainfall. Nature, 539(7629), 416–419. 10.1038/nature19819 27776357

[jgrd58205-bib-0035] Wang, M. , Xiao, M. , Bertozzi, B. , Marie, G. , Rörup, B. , Schulze, B. , et al. (2022). Synergistic HNO_3_–H_2_SO_4_–NH_3_ upper tropospheric particle formation. Nature, 605(7910), 483–489. 10.1038/s41586-022-04605-4 35585346 PMC9117139

[jgrd58205-bib-0036] Weigel, R. , Borrmann, S. , Kazil, J. , Minikin, A. , Stohl, A. , Wilson, J. C. , et al. (2011). In situ observations of new particle formation in the tropical upper troposphere: The role of clouds and the nucleation mechanism. Atmospheric Chemistry and Physics, 11(18), 9983–10010. 10.5194/acp-11-9983-2011

[jgrd58205-bib-0037] Williamson, C. J. , Kupc, A. , Axisa, D. , Bilsback, K. R. , Bui, T. , Campuzano‐Jost, P. , et al. (2019). A large source of cloud condensation nuclei from new particle formation in the tropics. Nature, 574(7778), 399–403. 10.1038/s41586-019-1638-9 31619794

[jgrd58205-bib-0038] Yanai, M. , Esbensen, S. , & Chu, J.‐H. (1973). Determination of bulk properties of tropical cloud clusters from large‐scale heat and moisture budgets. Journal of the Atmospheric Sciences, 30(4), 611–627. 10.1175/1520-0469(1973)030<0611:dobpot>2.0.co;2

[jgrd58205-bib-0039] Zimmerman, P. , Greenberg, J. , & Westberg, C. (1988). Measurements of atmospheric hydrocarbons and biogenic emission fluxes in the amazon boundary layer. Journal of Geophysical Research, 93(D2), 1407–1416. 10.1029/jd093id02p01407

