# Supplementary material for: The Role of Convective Up‐ and Downdrafts in the Transport of Trace Gases in the Amazon
Source: J Geophys Res Atmos. 2022 Sep 22;127(18):e2022JD037265. doi: 10.1029/2022JD037265 (PMC9787969; doi:10.1029/2022JD037265)
Supplement: Supplementary file 1 — Supporting Information S1 [file JGRD-127-e2022JD037265-s001.docx]

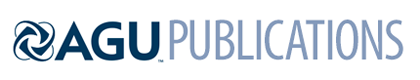


*JGR: Atmospheres*

Supporting Information for

**The role of convective up- and downdrafts in the transport of trace gases in the Amazon**

Roman Bardakov^1,2,3^ , Radovan Krejci^2,3^ , Ilona Riipinen^2,3^ , Annica M. L. Ekman^1,2^

^1^Department of Meteorology, Stockholm University, Stockholm, Sweden

^2^Bolin Centre for Climate Research, Stockholm University, Stockholm, Sweden

^3^Department of Environmental Science (ACES), Stockholm University, Stockholm, Sweden

**Contents of this file**

Figures S1 to S9

**Introduction**

This document contains supporting figures that were generated from simulations using the MISU-MIT Cloud-Aerosol Model (MIMICA) of deep convective clouds based on soundings retrieved in Manaus, Brazil from April 1 until April 14, 2020. The simulations were used to obtain air parcel trajectories for further calculations. The figures support analysis in the main text.


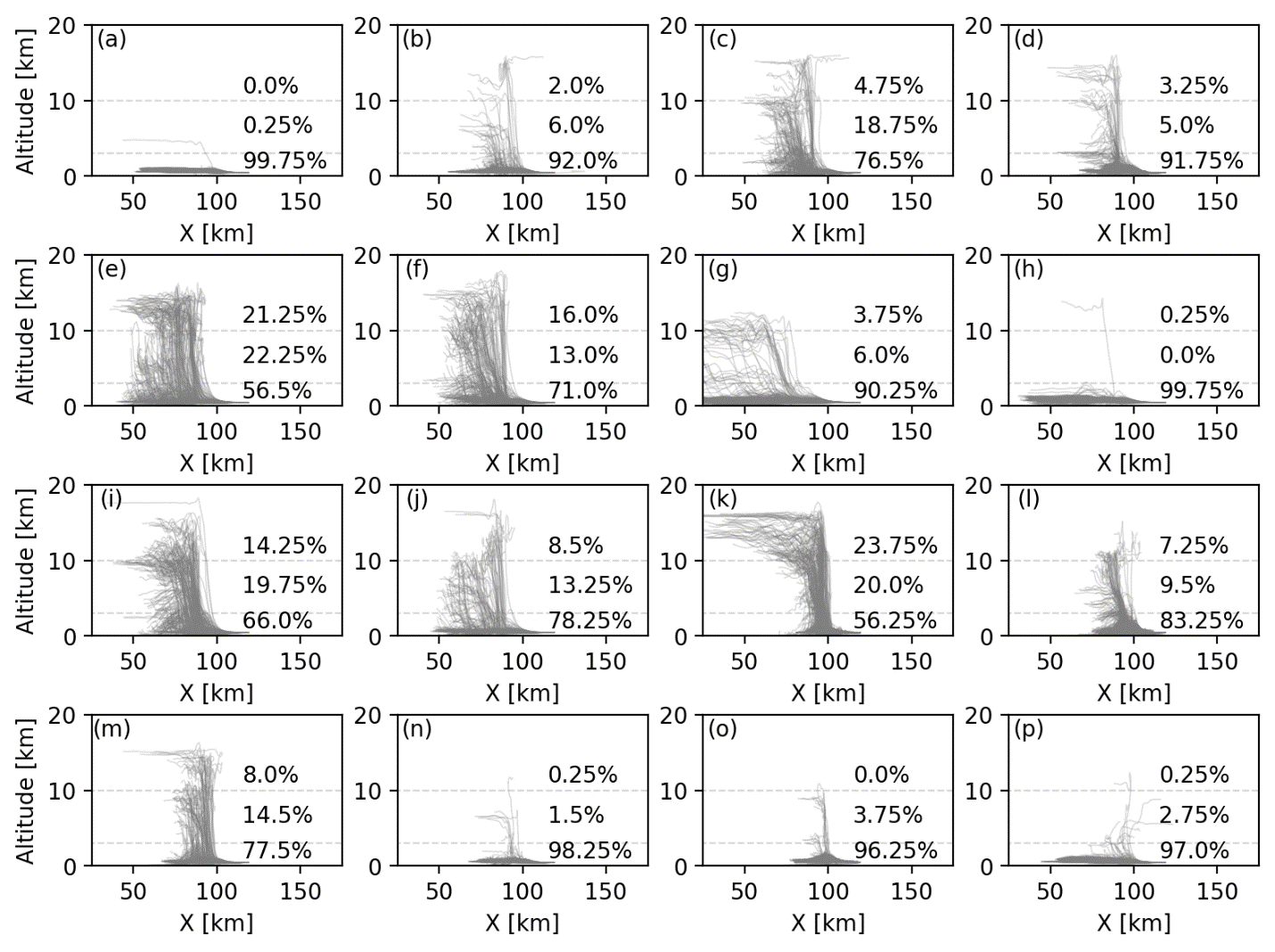


**Figure S1.** Trajectories for air parcels initially located at 0.5 km altitude for the deep convective cloud cases shown in Fig. 1 of the main text but for an initial maximum bubble perturbation of 3K. Top, middle and bottom numbers in each subfigure show percentages of parcels that reached the > 10 km, 3-10 km and 0-3 km height bins, respectively. The simulation time is 2 hours.


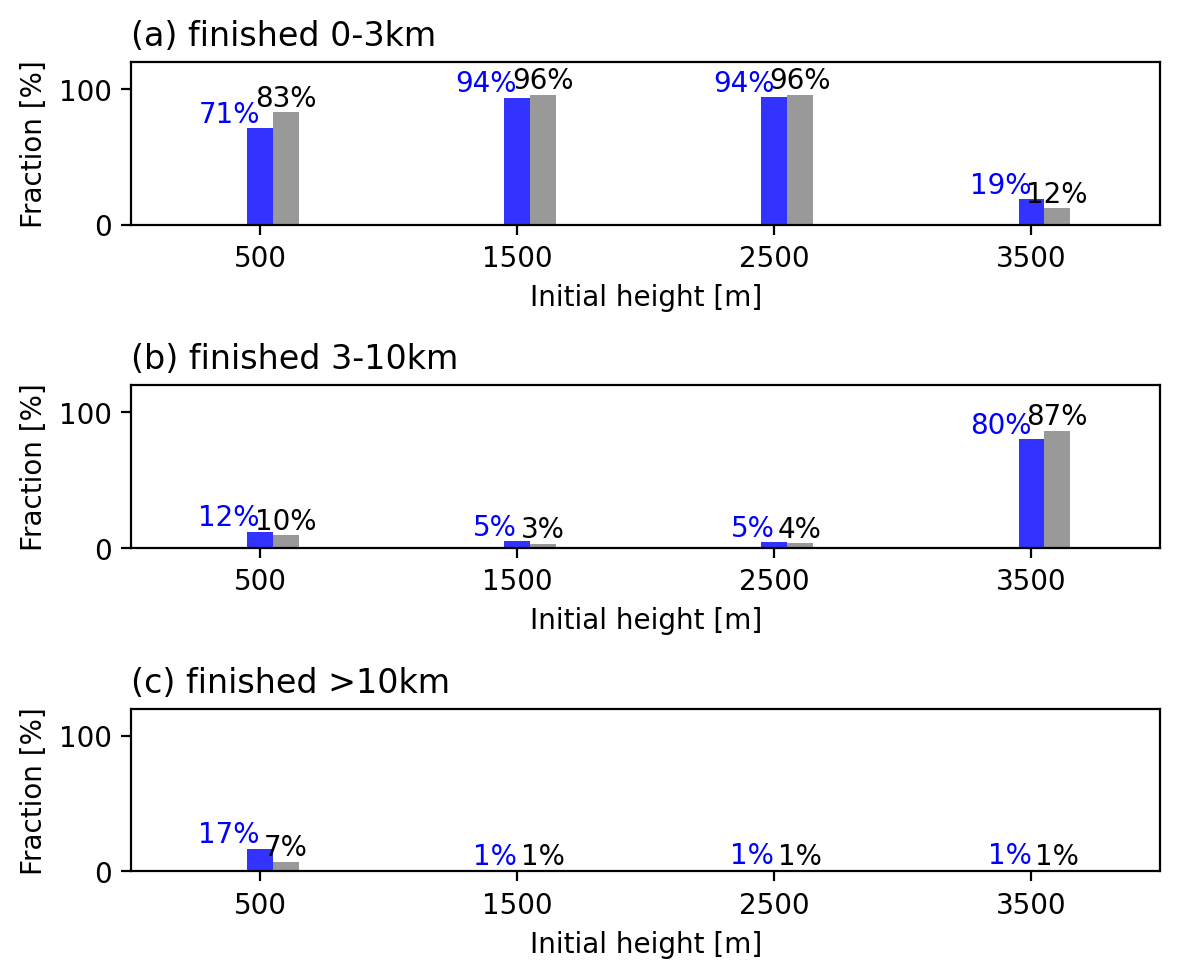


**Figure S2.** Fractions of parcels that end up within a specific height bin (BL: 0-3 km, middle troposphere: 3-10 km and UT: > 10 km, which represents the convective cloud outflow) after 2 h of simulations as a function of the initial parcel altitude. Blue color indicates fractions for a maximum initial bubble perturbation of 5K, gray indicates the fractions for a maximum initial bubble perturbation of 3K.


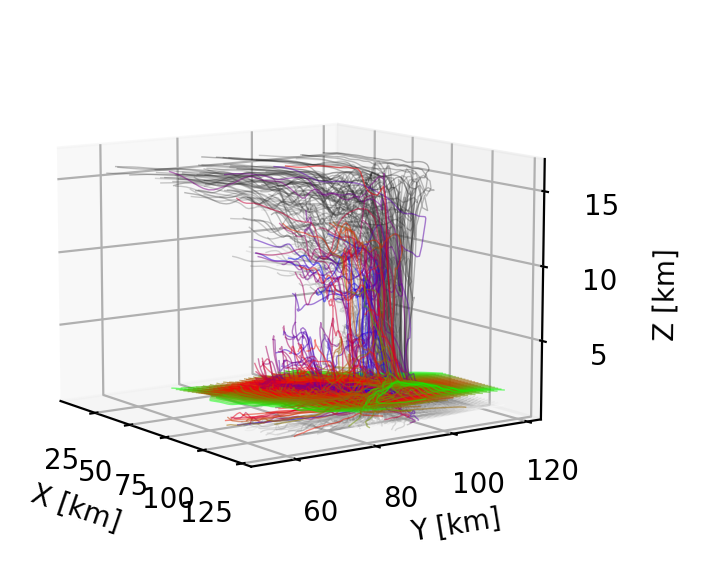


**Figure S3.** Three-dimentional air parcel trajectories of a deep convective cloud simulated based on the thermodynamic profile from April 9, 2020 (12 UTC), Manaus, Brazil. Grey trajectories start at 0.5 km height; blue-red-green trajectories start at 1.5 km. The trajectories are extracted after approximately 2 hours of simulation.


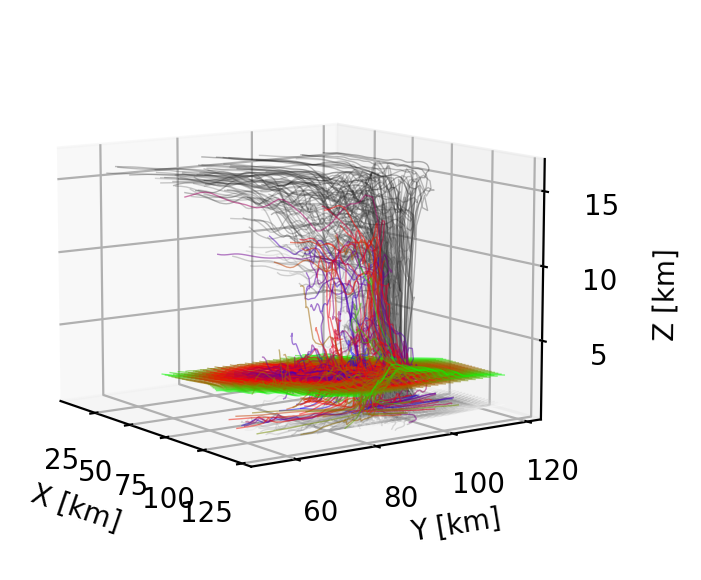


**Figure S4.** Same as in Fig. S3 but the blue-red-green trajectories start at 2.5 km.


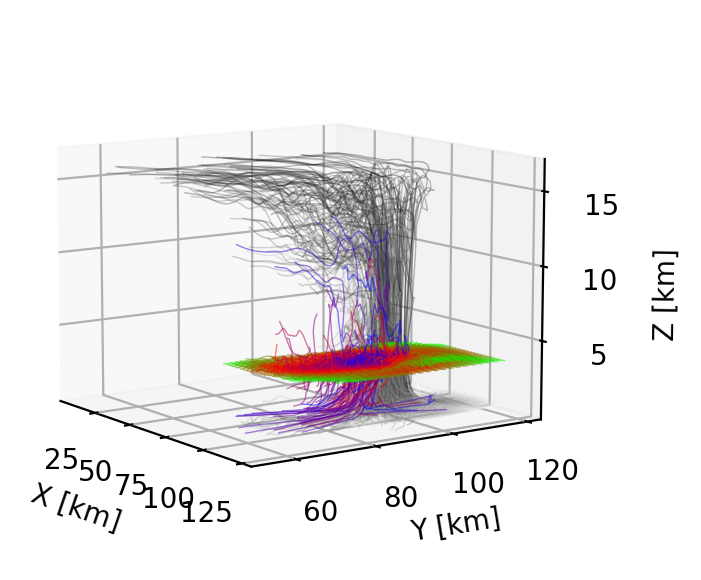


**Figure S5.** Same as in Fig. S3 but the blue-red-green trajectories start at 3.5 km.


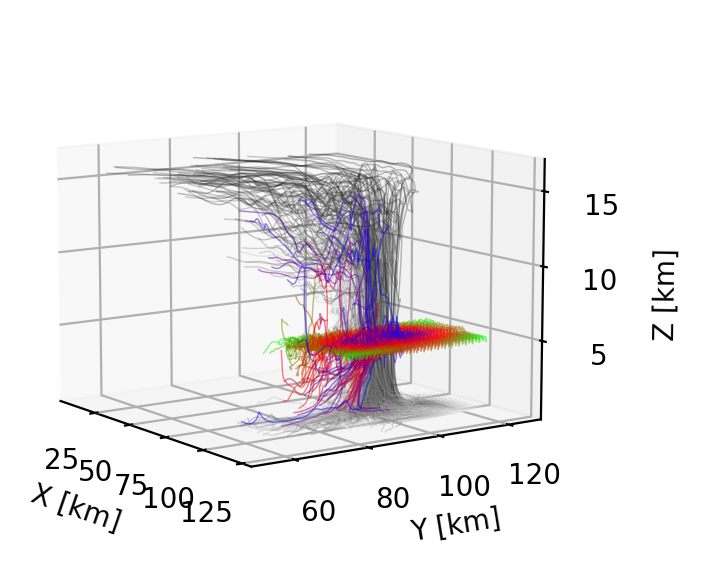


**Figure S6.** Same as in Fig. S3 but the blue-red-green trajectories start at 5 km.


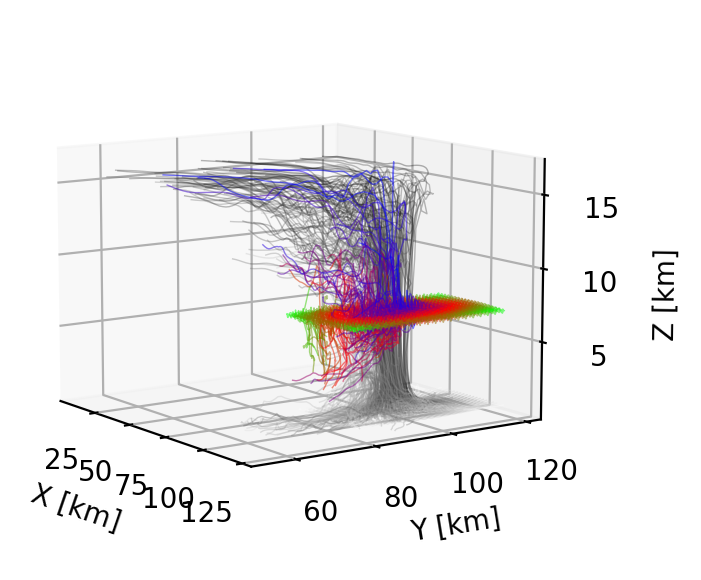


**Figure S7.** Same as in Fig. S3 but the blue-red-green trajectories start at 7 km.


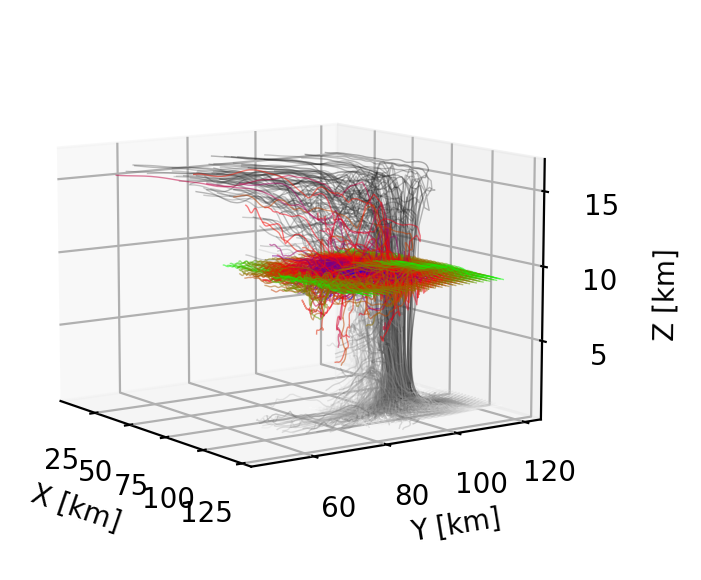


**Figure S8.** Same as in Fig. S3 but the blue-red-green trajectories start at 9 km.


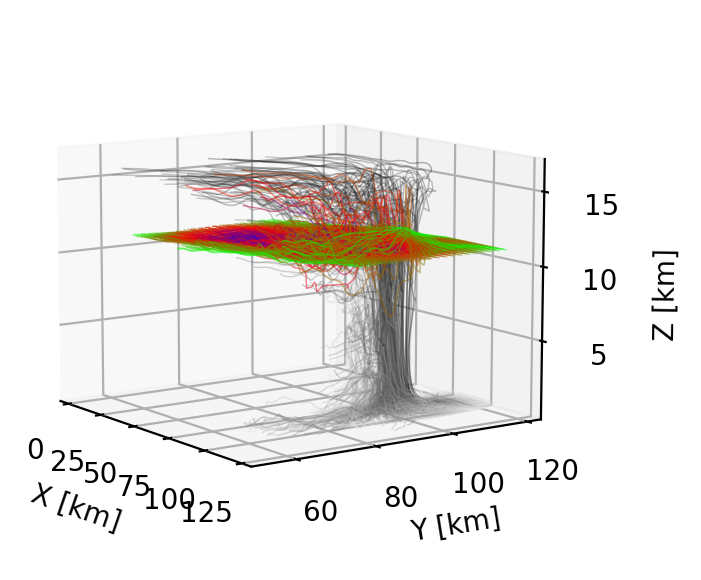


**Figure S9.** Same as in Fig. S3 but the blue-red-green trajectories start at 11 km.
